# Application of Multi‐Rod Constructs for the Revision of Thoracolumbar Fractures

**DOI:** 10.1111/os.14127

**Published:** 2024-07-09

**Authors:** Xiangchao Zeng, Yongwei Lv, Yafeng Yang, Xin Yin, Li Li, Huadong Wang, Ning Yu, Yan Wang, Jidong Guo

**Affiliations:** ^1^ Chinese PLA Medical School Beijing China; ^2^ Department of Orthopaedics Fourth Medical Center of Chinese PLA General Hospital Beijing China; ^3^ Department of Orthopaedics First Medical Center of Chinese PLA General Hospital Beijing China

**Keywords:** Multi‐rod, Renovation, Rod fracture, Sagittal Cobb angle, Thoracolumbar fracture

## Abstract

**Objective:**

The revision procedure for failure of internal fixation after thoracolumbar fracture is controversial. Combined anterior and posterior surgery is associated with higher risk more intraoperative bleeding and tissue damage. The success rate of simple anterior surgery needs further confirmation, and posterior surgery lacks stability of internal fixation. This study evaluates the feasibility and surgical effect of multi‐rod constructs in the revision of thoracolumbar fractures.

**Methods:**

Eleven patients with thoracolumbar fractures who underwent previous construct failure and were treated with revision and internal fixation with the multi‐rod technique from March 2017 to September 2018 were analyzed. The original internal fixation was removed and replaced in the medial insertion of satellite rods and bone graft. The average follow‐up time was 15.97 months. The intraoperation blood loss, the time of the operation, activation and discharge and the rate of rod fracture were calculated. The sagittal Cobb angle before revision, after revision and at the last follow‐up were compared. The clinical effect was evaluated by visual analogue scale (VAS) and Oswestry Disability Index questionnaire (ODI).

**Results:**

The average operation time was 107 min, the intraoperative blood loss was 131.81 mL, the active time was 1.59 days, and the discharge time was 10.89 days. No rod fractured again during the follow‐up period.

The paired *t*‐test was used to compare the Cobb angle, VAS score, and ODI before and after surgery. There was significant difference in the sagittal Cobb angle between the pre‐revision and the posterior sagittal position (*p* = 0.000), and no significant difference was found between post‐revision and last follow‐up (*p* = 0.551). VAS and ODI were greatly improved at the last follow‐up.

**Conclusion:**

The literature on revision of thoracolumbar fractures is insufficient and comprises varying opinions. This paper proposes a new treatment option. The application of the multi‐rod constructs in the revision of thoracolumbar fractures is safe, simple, and effective and might provide guidance for future clinical work.

## Graphical abstract

Multi‐rods technique can be used in the revision of fracture rod after operation of severe thoracolumbar fracture. After all, the operation is simple and easy, and the clinical outcome of short‐term follow‐up is good. In addition, there is no failure of internal fixation, and it is possible to avoid anterior revision surgery. The deficiency of this paper lies in the small sample size and short follow‐up time, and whether the multi‐rods technique can stand the test remains to be tested by time and more research.

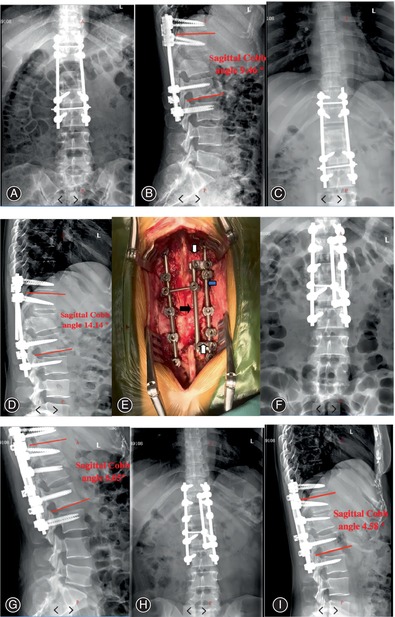

## Introduction

Posterior surgery has become the most used method for the treatment of thoracolumbar and lumbar fractures because of the advantages of being low risk and outstanding early postoperative outcome. However, the use of posterior internal fixation without spinal fusion or anterior column reconstruction for severe burst fractures or fracture dislocation will increase the risk of internal fixation failure especially in patients with long constructs.^1^ The common revision procedures for thoracolumbar fractures include one‐stage posterior replacement of internal fixation, resection of injured vertebra, reconstruction, internal fixation and posterior spinal decompression, bone graft fusion, and replacement of internal fixation.^2^, ^3^ Severe thoracolumbar fractures often destroy the three‐column structure of the spine, and it is relatively susceptible to instrumental loosening, fracture and other problems after surgery. Despite the above‐described complications, there are few reports detailing how to repair and treat them.^3^

The usual approach to revision is to replace the internal fixation with a stronger one to restore the physiologic curvature of the spine.^4^ Some scholars (e.g., Luca *et al*.)^5^ believe that posterior stabilization is insufficient and additional anterior reconstruction is necessary. Some scholars suggest that anterior revision surgery can provide excellent stability and play a role in preventing internal fixation failure.^6^ It has been suggested in the literature^7^, ^8^ that posterior internal fixation alone is satisfactory and can be performed without fusion. It has also been reported that patients with adjacent disc tissue damage are not recommended for posterior surgery.^9^ There is also some controversy about the choice of short‐segment or long‐segment internal fixation treatment when performing posterior surgery.

Some studies have proposed that the application of the satellite rod technique in three‐column osteotomy can reduce surgical trauma,^10^ result in a strong internal fixation outcome, restore spinal balance, and be well maintained during follow‐up. Through an *in vitro* biomechanical study, GodzikJ *et al*.^11^ concluded that the multi‐rod technique can reduce the stress of the long segment fixation rod in the lumbosacral junction, thus reducing the risk of internal fixation failure. By comparing the imaging findings of double‐rod fixation and multi‐rod fixation in 132 cases of three‐column osteotomy followed up for at least 2 years, Hyun determined that the multi‐rod technique is safe, simple and effective and can increase the stability of the three‐column osteotomy area, preventing the failure of internal fixation and the formation of symptomatic pseudarthrosis.^12^ Shen *et al*. used the multi‐rod technique in the correction of 36 cases of complex spinal deformities.^13^ After a follow‐up period of 2 years, it was found that the rate of rod fracture after surgery was 8.3%. Even if the rod was fractured, it did not cause symptoms or orthopaedic loss, so there was no need for revision. According to Shen *et al*., the use of the multi‐rod technique can effectively reduce the risk of failure of internal fixation after three‐column osteotomy for severe spinal deformity.^13^ For patients with three‐column structure destroyed thoracolumbar burst fractures or fracture dislocation, the best method for repair remains controversial. There are few reports on the application of the multi‐rod technique in thoracolumbar fracture revision. We retrospectively analyzed the data of 11 patients who underwent fracture rod revision after a thoracolumbar fracture operation with the multi‐rod technique from March 2017 to September 2018. By reviewing the literature, we hope to further analyze and demonstrate the feasibility of multi‐rod constructs for the revision of thoracolumbar fractures and to clarify the safety and effectiveness of the multi‐rod constructs through the analysis of this group of cases. In summarizing the technical points of the operation, we make suggestions for future clinical work.

## Methods

### Surgical Procedure

#### Preoperative Evaluation

X‐rays were performed for all patients before surgery, and computed tomography (CT) was performed if necessary.

#### Anesthetic Posture

Routine general anesthesia was performed in prone position.

#### Surgical Operation

**FIGURE 1:**
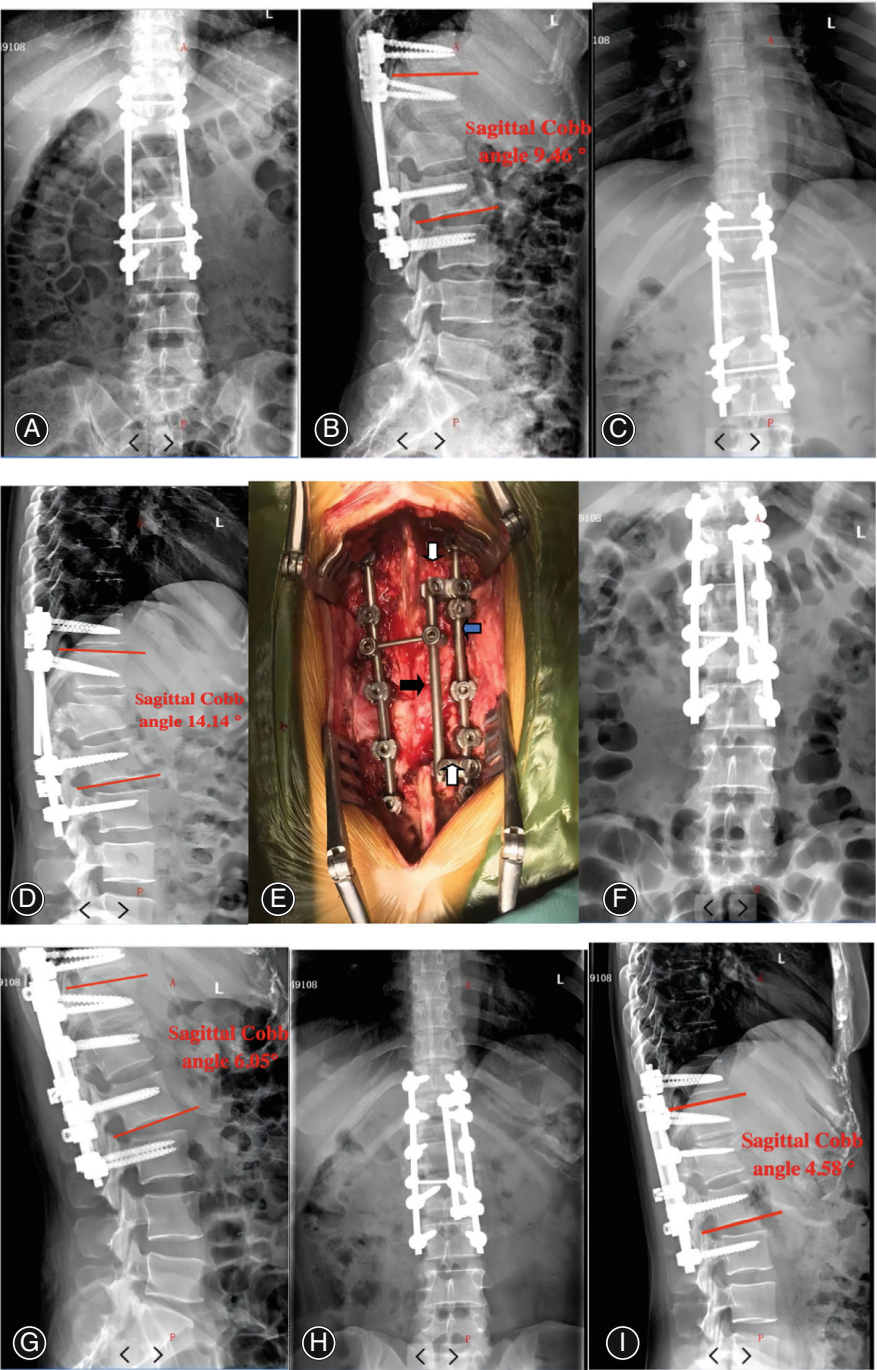
The patient was a 33‐year‐old woman with a lumbar 1 fracture and dislocation (ASIA A). (A, B) Positive and lateral position immediately after the first operation, sagittal Cobb angle 9.46°. (C, D) the left rod was fracture 12 months after operation, and the local sagittal Cobb angle was 14.14°. (E) The multi‐rod structure placed during the operation. The blue arrow refers to the replaced master rod, and the black arrow refers to the inner satellite rod. The white arrow refers to the connector between the master rod and the satellite rod. (F, G) Anterior and lateral films immediately after revision; sagittal Cobb angle 6.05°. (H and I) During the 1‐year follow‐up, the sagittal Cobb angle was 4.58°, with no fracture rod and no loss of kyphosis.

The internal fixation on the side of the fracture rod was exposed along the original incision. First, the osteophyte or fusion affecting the replacement of the rod was removed with osteotome. After removing the fracture rod, if we found that the screw was loose, we placed the screw if necessary and then reposition the cobalt‐chromium rod (Johnson, USA). In addition, a satellite rod (Figure 1E, Co–Cr rod, Johnson, USA) was reinserted on the inside, and the head and tail side of the satellite rod were connected with the original Co–Cr rod or the replaced Co–Cr rod with a connector (Figure 1E), respectively. Besides, the head and tail side needs to span at least one segment to form a strong and stable internal fixation. Finally, bone and BMPs was grafted interlaminar or between the transverse processes and the incision was closed.

#### Postoperative Treatment

Ankle pump exercises and straight leg raising training of both lower limbs were performed on the first day after operation. On the second day after the operation, patients were encouraged to get out of bed under the protection of brace. On the third day after the operation, professional rehabilitation physicians were asked to guide functional exercise. The motor function basically returned to the pre‐fracture level 3 months after operation.

#### Outcome Measures

The outcome measures included perioperative general condition, operative efficacy, and clinical effect. The operative time, intraoperative blood loss, ambulation time, and time to discharge were counted for perioperative general condition. Cobb angle is one of the commonly used outcome measures, especially for evaluating the surgical effect. The Cobb angle is measured as the angle between the adjacent upper endplate of the vertebral body and the perpendicular line of the parallel adjacent lower endplate of the vertebral body at the cephalic side of the injured vertebra. VAS was used for assessing thoracolumbar back pain, and ODI was used for assessing the effect of pain on daily life.

### Clinical Data and Statistical

#### General Information

Patients who underwent revision surgery for previous construct failure using the multi‐rod technique from March 2017 to September 2018 in our institution were statistically analyzed. The inclusion criteria included: (i) patients who had imaging evidence of a thoracolumbar fracture; (ii) patients who underwent posterior internal fixation surgery for thoracolumbar fracture; (iii) patients who experienced internal fixation failure after initial surgery; (iv) patients who received revision surgery with multi‐rod constructs; (v) patients with complete images, including images from the initial surgery and complete images before and after revision; and (vi) follow‐up should be at least 12 months. Exclusion criteria were as follows: (i) internal fixation failure due to surgical complications such as postoperative infection; (ii) patients who underwent other treatment rather than revision and internal fixation with the multi‐rod technique; and (iii) patients who failed the follow‐up assessment. This study was approved by the Institutional Review Board of our hospital (2021KY009‐HS001) and written informed consent was obtained from all patients participating in this study.

The average age was 35 ± 5.62 years old. According to AO classification,^14^ there were three cases of type C fracture and eight cases of type B fracture. There were four cases of L1 fracture, three cases of L2 fracture and four cases of L3 fracture. According to the American Spinal Injury Association (ASIA) classification, there were three cases of grade A, two cases of grade B, five cases of grade C, and one case of grade D at the initial injury. The first operation was performed with posterior intraspinal decompression and multi‐segmental pedicle screw–rod system internal fixation. The average time of rod breaking was 11.92 ± 3.44 months after surgery (Table 1).

**TABLE 1:** General Data of 11 Patients with Thoracolumbar Fractures.

Pt. No.	Age (years)	AO Fracture Classification	Fracture Site	ASIA Classification	Time of Rod Fracture
1	43	C	L3	A	14.6
2	32	B	L1	C	6
3	40	B	L3	B	9.7
4	37	C	L3	C	10.8
5	28	B	L2	B	18.5
6	33	B	L1	A	12
7	35	C	L3	C	11.4
8	25	B	L2	D	15.5
9	39	B	L1	C	12.4
10	41	B	L2	A	8.3
11	32	B	L1	C	11.9

#### Statistical Analysis

Statistical software SPSS (SPSS 21.0, SPSS, USA) was used for data analysis. The preoperative, operative, and postoperative data are presented as mean ± standard deviation. The paired *t*‐test was used to compare the Cobb angle, VAS score, and ODI before and after surgery. Statistical significance was set as *p* < 0.05.

### Preliminary Results and Complications

All the 11 patients’ operations went smoothly. The operation time was 107 ± 16.99 min, the intraoperative bleeding volume was 131.81 ± 69.11 mL, the ambulation time was 1.59 ± 0.45 days, and the discharge time was 10.89 ± 1.21 days. The follow‐up period was at least 1 year, with an average of 15.97 ± 3.67 months. As of the last follow‐up, no rod was fractured again after the operation, and there were no operation‐related complications.

#### Imaging Results and Typical Cases

**FIGURE 2:**
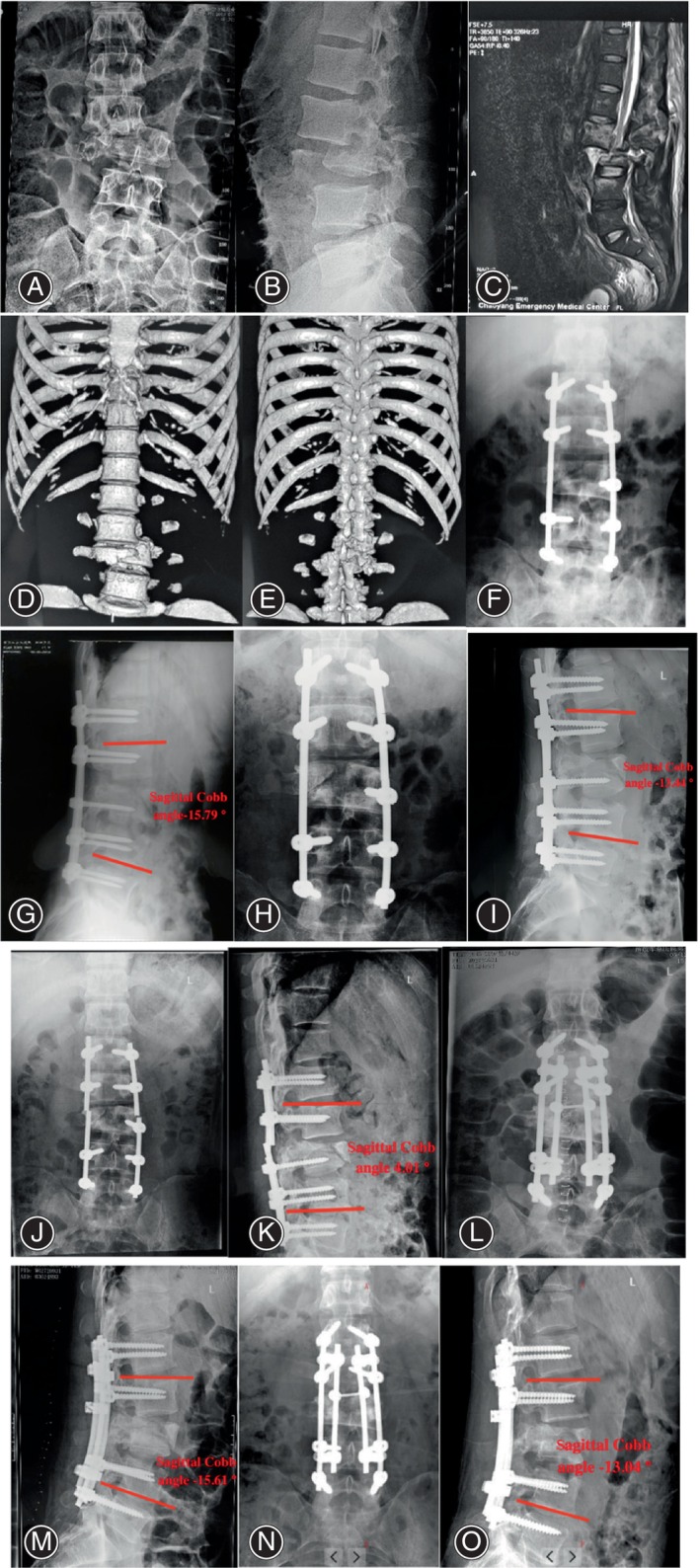
A 43‐year‐old man with lumbar 3 fracture and dislocation (ASIA A). (A–E) Preoperative X‐ray, CT, and MRI. (F, G) anterior and lateral position immediately after the first operation, sagittal Cobb angle −15.79°; (H, I) the left side was fractured 11 months after the first operation; the sagittal Cobb angle was –13.44°. (J, K) the right side was fractured 14 months after the first operation. Sagittal Cobb angle was 4.01°. (L, M) the sagittal Cobb angle was −15.61° immediately after revision; (N, O) during the 2‐year follow‐up, the sagittal Cobb angle was −13.04°, no fracture rod and no loss of kyphosis.

The Cobb angle is one of the commonly used parameters in spine surgery. The Cobb angle is measured as the angle between the adjacent upper endplate of the vertebral body and the perpendicular line of the parallel adjacent lower endplate of the vertebral body at the cephalic side of the injured vertebra. After surgical treatment, the sagittal Cobb angle was improved from 9.89° ± 3.19° to −1.93° ± 7.11°, and the difference was significant (*p* = 0.000). At the last follow‐up, the sagittal Cobb angle was −1.68° ± 5.82°, which was not different from that immediately after revision (*p* = 0.551). Typical cases are shown as Figures 1 and 2.

#### Clinical Effect

Non‐steroidal anti‐inflammatory drugs were routinely administered for postoperative analgesia. The VAS score decreased from 6.27 ± 1.27 before revision to 1.64 ± 0.67 at the last follow‐up.^15^ In addition, the ODI score^16^ decreased from 38.36 ± 6.17 before revision to 8.55 ± 2.30 at the last follow‐up. The function of the patient gradually improved, and there was no rod fracture at the last follow‐up.

## Discussion

Multi‐rod structures have been widely used due to the superior posterior stability they provide. Reviewing the literature, we found that the multi‐rod construct is highly feasible for use in the revision of thoracolumbar fractures. The retrospective analysis of the cases demonstrates that multi‐rod constructs have the advantage of being less invasive, safe, and effective in revision of thoracolumbar fracture. The combination of multi‐rod constructs with screw placement in fracture vertebral and posterolateral bone grafting can achieve a satisfactory surgical outcome. Based on the review of the above cases, we determined that the multi‐rod construct revision of thoracolumbar fractures internal fixation failure has the advantages of short operation time and less bleeding and reduced ambulation time and hospital stay. After surgery, patients’ thoracolumbar curvature improved satisfactorily, and the VAS score and ODI were significantly improved.

1. Feasibility of the multi‐rod constructs for the revision of thoracolumbar fracture

The multi‐rod technique was first used in lumbar–pelvic reconstruction after total sacral resection of sacral chordoma by Shen^17^ in 2006. The authors used a four‐rod technique to reconstruct the lumbar–pelvic connection based on the patient's obese condition and his willingness to ambulate at an early stage. No failure of internal fixation was found in early follow‐up. Seyed Vosoughi,^18^ in a spine–pelvic finite element analysis, illustrated that the satellite rod can not only improve the stability and reduce the range of motion by increasing the stiffness of the internal fixator but also reduce the stress of the original rod in the osteotomy area and transfer the stress from the osteotomy area to the adjacent area of the connector. In terms of reducing the stress of the original rod in the osteotomy area, the medial satellite rod is significantly better than the lateral satellite rod. In addition, the medial satellite rod does not affect the lateral bone graft, nor does it affect the closure of the incision. The application value of multi‐rod technology is related not only to the position of the satellite rod but also to the material and diameter of the rod. Through the biomechanical study of five cadaveric specimens that had undergone lumbar pedicle osteotomy, Hallager *et al*.^19^ concluded that all the screw–rod structures could significantly reduce the flexion and extension and left and right flexion in the osteotomy area. However, only the Co–Cr rod could significantly reduce the axial rotation. Compared with the two‐bar technology of titanium alloy, the four‐rod technology of cobalt–chromium can reduce the stress of the original bar by 65%. The operation of four titanium rods combined with interbody fusion can reduce the stress of the original rod by 62%. The cobalt–chromium four‐bar technique combined with interbody fusion can provide the strongest mechanical stability and reduce the original bar stress by 76%. Through finite element analysis, Luca^20^ also suggested that the Co–Cr four‐rod construct can most effectively reduce the stress on the fixation in the osteotomy area. It is considered that the longitudinal connecting rod with a larger diameter can reduce the risk of internal fixation failure. This view is consistent with the proposition of Smith.^21^ The above literature shows that the use of the multi‐rod technique in spinal three‐column osteotomy can achieve the purpose of stress dispersion, better maintenance of mechanical stability of the osteotomy area, and reduction in the failure of internal fixation.

A review of the literature revealed that the main reported causes of internal fixation failure in thoracolumbar fractures include: anterior fusion failure, misplacement of the posterior pedicle screw, re‐fracture of the adjacent vertebra, over distraction during the first operation, and improper implant removal.^22^ We believe that the primary cause of internal fixation failure is poor strength of the internal fixation, which fails to provide a solid fusion, and is also caused by incorrect procedures during the initial surgery, such as overstretching. Therefore, it is important to increase the strength of the internal fixation during revision surgery.

For the thoracolumbar fracture with a severely damaged of three‐column structure, what kind of revision operation should be taken when the rod is fractured? In our opinion, it is necessary to recognize the mechanical mechanism of rod breakage. In severe thoracolumbar fractures, the anterior, middle, and even three columns of the spine are often seriously damaged. In addition, the anterior and middle columns of the spine are not reconstructed during posterior internal fixation. Nonunion or delayed union of the fracture can impose substantial stress on the posterior internal fixation due to the significant loss in mechanical stability in the anterior and middle spinal colunms. This can easily lead to internal fixation failure and kyphosis. It remains controversial whether fusion or reconstruction of the anterior middle column is needed after internal fixation and distraction reduction of a spinal fracture. Some studies^7^, ^8^ have suggested that the curative effect of simple posterior internal fixation is satisfactory without additional anterior fusion. However, it is also reported that the reconstruction of the anterior and middle column of the spine is a key measure to prevent the failure of internal fixation and kyphosis after a thoracolumbar burst fracture.^2^, ^3^

In our study, 11 cases of spinal fractures were treated with posterior intraspinal decompression and multi‐segmental pedicle screw–rod system internal fixation without the anterior and middle column of the spine reconstructed. The rod was fractured approximately 11.92 months after the operation and the fractured vertebral body did not heal when the rod was fractured. We speculate that the fracture rod might be related to the failure to reconstruct the anterior and middle column of the spine and the nonunion of the fractured vertebral body. In view of these cases, some scholars believe that the anterior middle column of the spine must be reconstructed. This undoubtedly increases surgical complications such as excessive bleeding and nerve injury. We believe that the simple posterior approach is worth a try to reduce the risk of surgical trauma and nerve injury and avoid anterior surgery. The multi‐rod technology can provide good mechanical stability, achieve the purpose of stress dispersion, and reduce likelihood of internal fixation failure. In addition, in revision cases, the front instability will not be as serious as when the injured occurred due to factors such as scars. There is hope of achieving a solid fusion with posterior‐only surgery. A related biomechanical study^17^, ^23^ showed that the four‐rod technique, with the rod made of cobalt–chromium, is equivalent to using four titanium rods combined with interbody fusion to reduce the stress of the original rod. This also provides a theoretical basis for us to use only posterior multi‐rod fixation in the revision of the fracture rod after a spinal fracture.

2 Effectiveness of the multi‐rod constructs for the revision of a thoracolumbar fracture

Eleven cases of thoracolumbar fracture were treated with posterior multi‐rod fixation because the multi‐rod technique can disperse the stress, maintain the mechanical stability of the osteotomy area, and reduce the failure of internal fixation. The average operation time was approximately 107 min, and the average intraoperative blood loss was approximately 131.81 mL. The time of ambulation was approximately 1.59 days, and the time of discharge was approximately 10.89 days. This shows that the multi‐rod technique is superior to the first‐stage anterior and posterior revision in terms of operation time, intraoperative blood loss, field time, and discharge time. The average follow‐up time was 15.97 months. There was no failure of internal fixation such as a fractured screw, a fractured rod, or screw–rod loosening. There was no loss of orthopaedics at the last follow‐up. Multi‐rod technology can provide strong mechanical stability and minimize the risk of internal fixation failure. In this study, after spinal fracture, the fracture rod caused low back pain, which was not relieved after conservative treatment, such as the use of medicine and physiotherapy, and seriously affected the functional exercise. The VAS score and ODI score at the last follow‐up after multi‐rod fixation were significantly improved compared with those before revision. These results demonstrate that the application of the multi‐rod technique in the revision of the fracture rod after spinal fracture can significantly improve the clinical symptoms and quality of life of patients. In addition, in patients with severely three‐column structure injuries, we attempt to use multi‐rod fixation in the first stage to obtain shorter fixed segments and more rigid fixation.

3 Technical points

First, the main rod is placed and then the medial satellite rod, which does not affect the lateral bone grafting and incision closure. The medial satellite rod is significantly better than the lateral satellite rod in reducing the stress of the original rod in the osteotomy area. The radian of the satellite rod is consistent with that of the ipsilateral master rod. The head and tail of the satellite rod needs to span at least one segment each to form a strong and stable internal fixation.

In addition to the multi‐rod technique, screw placement is performed when the pedicle is intact. It contributes to reconstruct the spinal sequence and increase the mechanical stability of the posterior internal fixation system.^24^ There are many factors affecting bone healing, among which biomechanics is more important. In the process of fracture healing, excessive stress will lead to fracture instability, resulting in delayed union or nonunion. Conversely, internal fixation being too solid can cause too little stress at the fracture site, which might not be enough to stimulate bone formation. As a consequence, we apply rhBMP‐2, at the same time of posterolateral bone grafting, which might be beneficial to fusion.

### Prospects of Clinical Application

The anatomical and mechanical factors of the thoracolumbar result in susceptibility to fracture. Most of the studies focus on initial surgery for fractures and there is a lack of research on revision surgery. Among these studies, anterior surgery, posterior surgery, and combined anterior and posterior surgery are controversial. Multi‐rod constructs for the revision of thoracolumbar fracture have the advantages of causing less trauma, shorter operating time, less surgical bleeding, and safe and effective surgical efficacy, which can provide a new treatment option for clinical work and further improve the research on the revision of thoracolumbar fractures. In addition because such structures can provide strong internal fixation support, they can also be used for the treatment of Andersson lesions and for surgery involving L5/S1 segments. Related studies are also in progress.

There are also some potential risks associated with this surgical procedure, such as non‐union of the fracture, increased implants, and increased risk of infection.

### Limitations

Due to the limited number of cases in this group and the absence of long term follow‐up data for some patients, who either passed away or were lost to follow‐up, we are planning a large‐scale, muti‐center study to gather more dependable data.

## Conclusion

The multi‐rod technique can be used in the revision of fracture rods after operations for severe thoracolumbar fracture. The operation is simple and easy, and the clinical outcome based on short‐term follow‐up is good. In addition, there is no failure of internal fixation, and it is possible to avoid anterior revision surgery.

## Author Contributions

All authors have read and approved the final manuscript. XCZ was a major contributors in designing the study and writing the manuscript. JDG and YW were involved in the study design, analysis, and interpretation of the data, and drafting and revising of the manuscript. YWL and NY were involved in the data collection. LL and HDW were involved in the analysis and interpretation of data. All authors read and approved the final manuscript.

## Ethics Statement

Approval was obtained from the ethics committee of the Fourth Medical Center of PLA General Hospital (2021KY009‐HS001). The procedures used in this study adhere to the tenets of the Declaration of Helsinki.

## Conflict of Interest Statement

The authors declare no conflict of interest.
